# Supplementary material for: Extracellular Vesicle Treatment Alleviates Neurodevelopmental and Neurodegenerative Pathology in Cortical Spheroid Model of Down Syndrome
Source: Int J Mol Sci. 2023 Feb 9;24(4):3477. doi: 10.3390/ijms24043477 (PMC9960302; doi:10.3390/ijms24043477)
Supplement: Supplementary file 1 [file ijms-24-03477-s001.zip › ijms-2147840-supplementary.pdf]

Supplemental Figure S1

PHF13

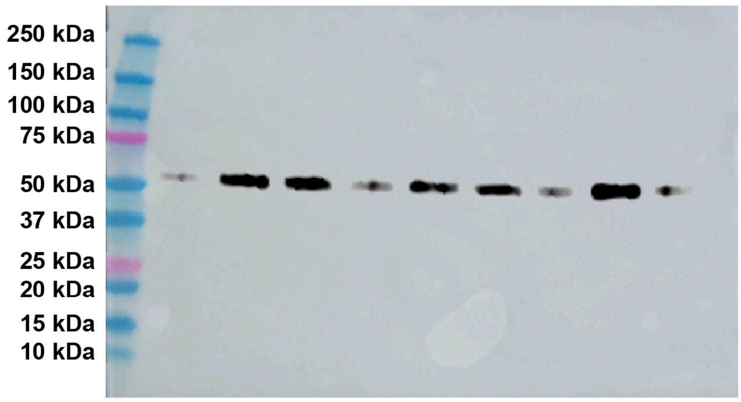

Actin

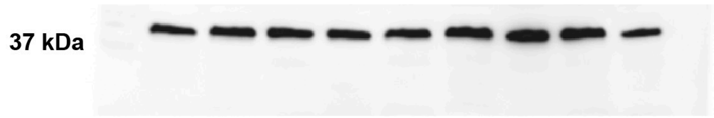

Supplemental Figure S1. Western blot raw images corresponding to Figure 7A visualized with PHF13 and actin antibodies in whole homogenates of euploid, trisomic, and EV treated trisomic CS. Actin image is overexposed in order to show the 37 kDa marker.
